# The E3 ubiquitin ligase activity of RING1B is not essential for early mouse development

**DOI:** 10.1101/gad.268151.115

**Published:** 2015-09-15

**Authors:** Robert S. Illingworth, Michael Moffat, Abigail R. Mann, David Read, Chris J. Hunter, Madapura M. Pradeepa, Ian R. Adams, Wendy A. Bickmore

**Affiliations:** Medical Research Council Human Genetics Unit, Institute of Genetics and Molecular Medicine, University of Edinburgh, Edinburgh EH42XU, United Kingdom

**Keywords:** PRC1, histone ubiquitination, H3K27me3, embryonic stem cell, Polycomb

## Abstract

Polycomb-repressive complex 1 (PRC1) and PRC2 maintain repression at many developmental genes in mouse embryonic stem cells and are required for early development. However, it is still unclear how they are targeted and how they function. We show that the ability of RING1B, a core component of PRC1, to ubiquitinate histone H2A is dispensable for early mouse embryonic development and much of the gene repression activity of PRC1. Our data support a model in which PRC1 and PRC2 reinforce each other's binding but suggest that the key functions of PRC1 lie beyond the enzymatic capabilities of RING1B.

## Abstract (precis)

Illingworth et al. show that the ability of Ring1B, a core component of PRC1, to ubiquitinate histone H2A is dispensable for early mouse embryonic development and much of the gene repression activity of PRC1.

There are two principal types of Polycomb group (PcG) complexes. Polycomb-repressive complex 2 (PRC2) is responsible for trimethylation of Lys27 on histone H3 (H3K27me3) via the EZH2 or EZH1 protein subunit (Di Croce and Helin 2013). Canonical PRC1 contains CBX subunits (the vertebrate homologs of *Drosophila* Polycomb) whose chromodomains are able to bind H3K27me3 (Kaustov et al. 2011). PRC1 also contains the heterodimeric E3 ligase RING1B/PCGF1–6, which can catalyze the ubiquitination of Lys119 on histone H2A (H2AK119ub). The canonical form of PRC1 contains PCGF2 or PCGF4 (MEL18 or BMI1). More recently, other RING1B-containing complexes have been identified that lack CBX subunits and instead contain RYBP or its homolog, YAF2 (Gao et al. 2012; Tavares et al. 2012; Morey et al. 2013). These noncanonical PRC1 complexes can contain a variety of PCGF subunits.

While a role for H3K27me3 in the recruitment of PRC1 to chromatin is well established, more recently it has also been suggested that PRC1-mediated H2AK119ub is sufficient to recruit PRC2 in at least some contexts (Blackledge et al. 2014; Cooper et al. 2014; Kalb et al. 2014), thereby providing a mechanism by which PRC1 and PRC2 may cooperatively reinforce each other's respective binding. On the other hand, rescue of *Hox* gene repression by ectopic expression of a catalytically inactive RING1B in *Ring1B*-null mouse embryonic stem cells (mESCs) suggested that the repressive (and chromatin compaction) activities of canonical PRC1 may be largely independent of RING1B-mediated H2A ubiquitination (Eskeland et al. 2010), at least for classical polycomb targets such as *Hox* loci. However, in the absence of the RING1B paralog RING1A, expression of catalytically inactive RING1B in mESCs was reported to only partially rescue polycomb target gene repression (Endoh et al. 2012).

There is therefore considerable uncertainty about the role of RING1B catalytic function in polycomb-mediated repression and about the interrelationship between H3K27me3 and H2AK119ub. The in vivo role of RING1B's catalytic function has not been assessed.

By generating a mouse model that expresses endogenous RING1B with no H2A ubiquitination activity, we show that, in addition to rescuing the majority of gene misregulation exhibited by *Ring1B* knockout (*Ring1B*^−/−^) mESCs, catalytically inactive RING1B also permits development to progress much further than in *Ring1B*-null mice (Voncken et al. 2003). We conclude that although RING1B is essential for early murine embryonic development, its catalytic activity is not.

## Results and Discussion

### RING1B catalytic activity is dispensable for repression at most PRC1 target loci in mESCs

To determine the role of endogenous RING1B's E3 ligase activity, we generated a knock-in allele that expresses a mutant form of RING1B protein with an alanine at position 53 in place of isoloeucine (*Ring1B*^*I53A*^). This amino acid change has been shown to disrupt the interaction of RING1B with the E2 UBCH5C and ablates the ability of RING1B to act as an E3 ligase in vitro (Buchwald et al. 2006). However, I53A does not perturb the incorporation of RING1B into canonical and variant PRC1 complexes (Illingworth et al. 2012).

**Figure 1.**
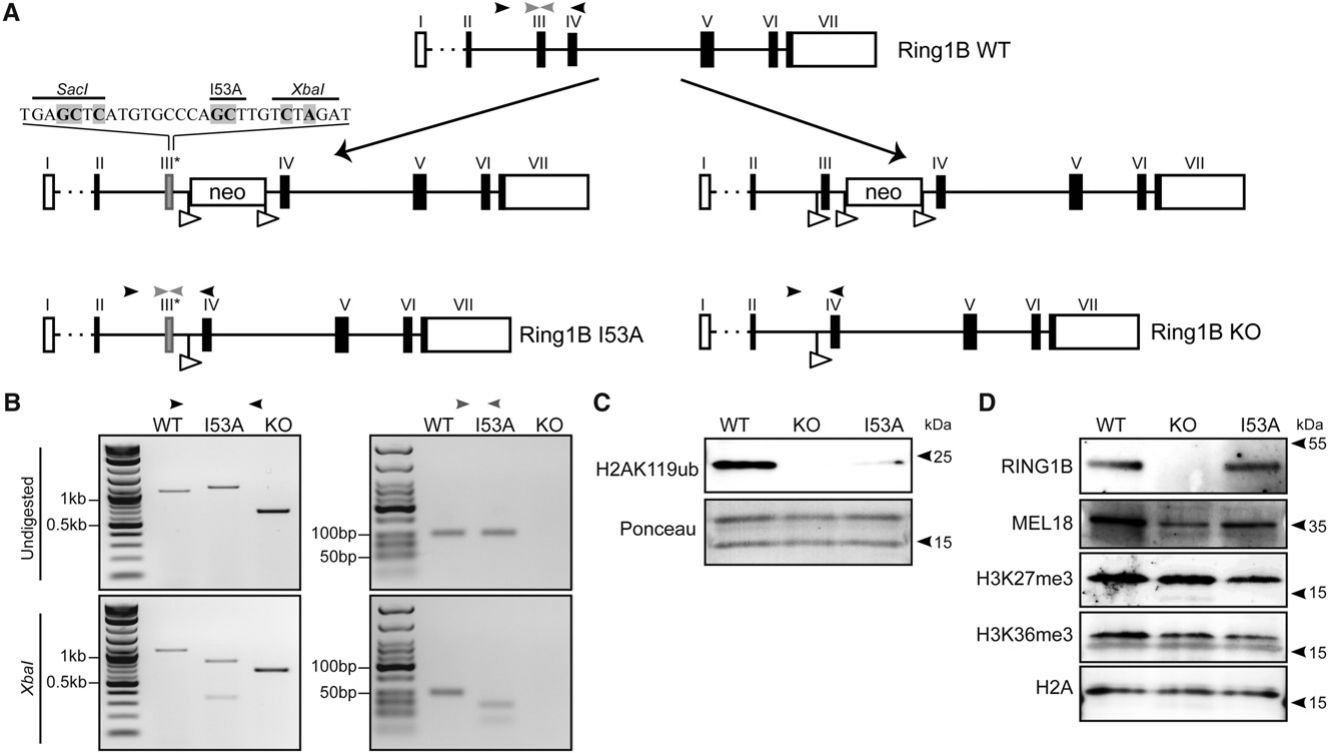
Targeting and validation of *Ring1B*^*I53A/I53A*^ and *Ring1B*^−/−^ mouse ESCs. (*A*) Schematic representation of the targeting strategy used to generate *Ring1B*^*I53A/I53A*^ and *Ring1B*^−/−^ (knockout [KO]) alleles. The locations of exon 3 internal and exon 3-spanning primer sites used for genotyping are indicated with gray and black arrowheads, respectively. I53A-specific sequence modifications introduced into exon 3 are shown shaded in gray, and LoxP sites are shown as open arrowheads. (*B*) Genotyping PCR results for the exon 3-spanning (*left* panel) and exon 3 internal (*right* panel) primer sets show the expected undigested (*top* panel) and XbaI-digested (*bottom* panel) profiles for each allele. (*C*,*D*) Immunoblotting of acid-extracted histones (*C*) and nuclear extracts (*D*) from wild-type (WT), *Ring1B*^−/−^ (knockout), and Ring1B^I53A/I53A^ (I53A) mESC lines for RING1B, MEL18, H3K27me3, H3K36me3, H2A, and H2AK119ub. Ponceau-stained histones (*C*) and H2A immunoblotting (*D*) served as loading controls.

Using homologous recombination, we generated heterozygous (*Ring1B*^+/*I53A*^) and homozygous (*Ring1B*^*I53A/I53A*^) knock-in alleles at the endogenous *Ring1B* locus in E14TG2a mESCs (Fig. 1A,B). The resulting cells are distinct from those generated previously (Eskeland et al. 2010) in that the mutation is introduced within the *Ring1B* coding sequence rather than as a transgene and therefore better preserves endogenous *Ring1B* expression levels. For direct comparison, we also derived *Ring1B*^−/−^ mESCs from the same parental E14TG2a mESCs (Fig. 1A,B). Immunoblotting showed a major loss of H2AK119ub in *Ring1B*^*I53A/I53A*^ and *Ring1B*^−/−^ mESCs, confirming the ablation of RING1B catalytic activity and a minor role for other E3 ligases, including RING1A, in maintaining H2AK119ub levels in these cells (Fig. 1C; van der Stoop et al. 2008; Zhou et al. 2008; Luijsterburg et al. 2012; Bhatnagar et al. 2014). *Ring1B*^*I53A/I53A*^ mESCs express RING1B protein at levels similar to wild-type (Fig. 1D) and appear to maintain levels of the canonical PRC1 component MEL18. Quantitative immunoblotting confirmed this while also showing a moderate reduction in the level of the noncanonical subunit RYBP (Supplemental Fig. 1). Conversely, *Ring1B*^−/−^ cells show a marked reduction in MEL18 levels, compatible with the destabilization of core PRC1 components in cells lacking RING1B (van der Stoop et al. 2008; Eskeland et al. 2010).

Despite the proposed mechanism by which H2AK119ub facilitates the deposition of H3K27me3 (Blackledge et al. 2014; Cooper et al. 2014; Kalb et al. 2014), we found that loss of H2AK119ub in *Ring1B*^−/−^ or *Ring1B*^I53A/I53A^ cells did not result in global reduction in H3K27me3 levels (Fig. 1D; Supplemental Fig. 1). Moreover, we did not detect an increase in H3K36me3 despite the proposed antagonistic relationship between H2AK119ub and H3K36me2/3 deposition (Fig. 1D; Yuan et al. 2013).

**Figure 2.**
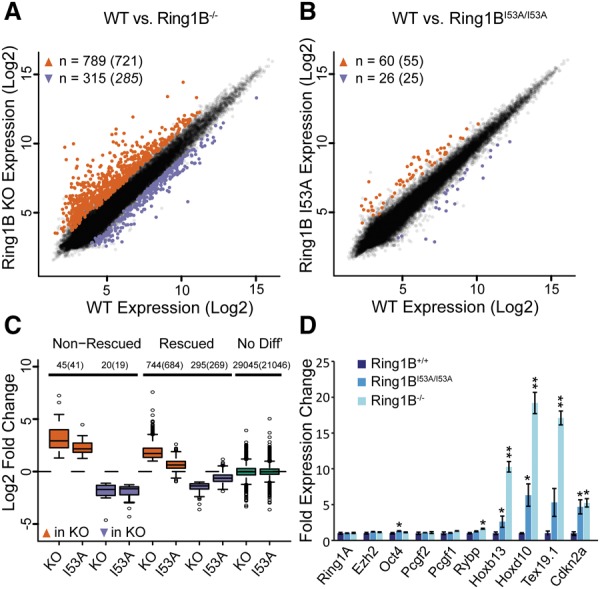
The E3 ligase activity of RING1B is largely dispensable for gene repression. (*A*,*B*) Log_2_ expression values in wild-type (WT) versus *Ring1B*^−/−^ mESCs (*A*) and *Ring1B*^*I53A/I53A*^ mESCs (*B*) from expression microarrays. Probes were considered to be up-regulated (orange) or down-regulated (blue) if they had a log_2_ fold change of >1 or less than −1, respectively, and a Benjamini-Hochberg-corrected *P*-value of <0.05. The number of differentially expressed probes and the number of genes they represent (in parenthesis) are indicated. (*C*) Box plots of log_2_ fold expression changes for *Ring1B*^−/−^ versus wild type (knockout [KO]) and *Ring1B* ^I53A/I53A^ versus wild type (I53A) for genes with “rescued” and “nonrescued” expression levels in *Ring1B*^*I53A/I53A*^ mESCs. The number of probes (genes in parentheses) is indicated for each subset. (*D*) Candidate expression analysis by quantitative RT–PCR. Plots show the mean expression across three biological replicates, with error bars indicating standard deviation. Significant differential gene expression, as determined by a Student's *t*-test, is indicated with asterisks. (*) *P*-value < 0.05 and > 0.01; (**) *P*-value < 0.01.

Using microarrays, we compared the expression profiles of *Ring1B*^−/−^ and *Ring1B*^*I53A/I53A*^ mESCs with wild type (Fig. 2A,B; Supplemental Table 1). Loss of RING1B results in hundreds of genes showing both significant up-regulation (721) and down-regulation (285) by more than twofold relative to wild type. Most of these changes are likely indirect, since, for those genes that are directly bound by RING1B in wild type, only 98 are up-regulated and 18 are down-regulated in knockout cells (Supplemental Fig. 2A,B). These changes were largely rescued in *Ring1B*^*I53A/I53A*^ cells where only 55 and 25 genes (12 and two RING1B-bound genes) showed up-regulation and down-regulation, respectively. Differentially expressed genes in *Ring1B*^*I53A/I53A*^ overlap well (41 of 55 up-regulated and 19 of 25 down-regulated) with those also showing differential expression in *Ring1B*^−/−^ mESCs. Even for this small number of “nonrescued” genes, the fold change in up-regulation relative to wild type is lower in *Ring1B*^*I53A/I53A*^ than in *Ring1B*^−/−^ (Fig. 2C; Supplemental Fig. 2C). Nonrescued genes were generally those with the highest level of up-regulation in *Ring1B*^−/−^ cells. Gene expression changes were confirmed by real-time RT–PCR (Fig. 2D). These data suggest that many of the “rescued” genes are still misregulated in *Ring1B*^*I53A/I53A*^ cells, but to a much lower extent, and hints that RING1B-mediated gene regulation is enhanced by, but not primarily dependent on, its catalytic activity.

### RING1B and H3K27me3 deposition is impaired in I53A cells

**Figure 3.**
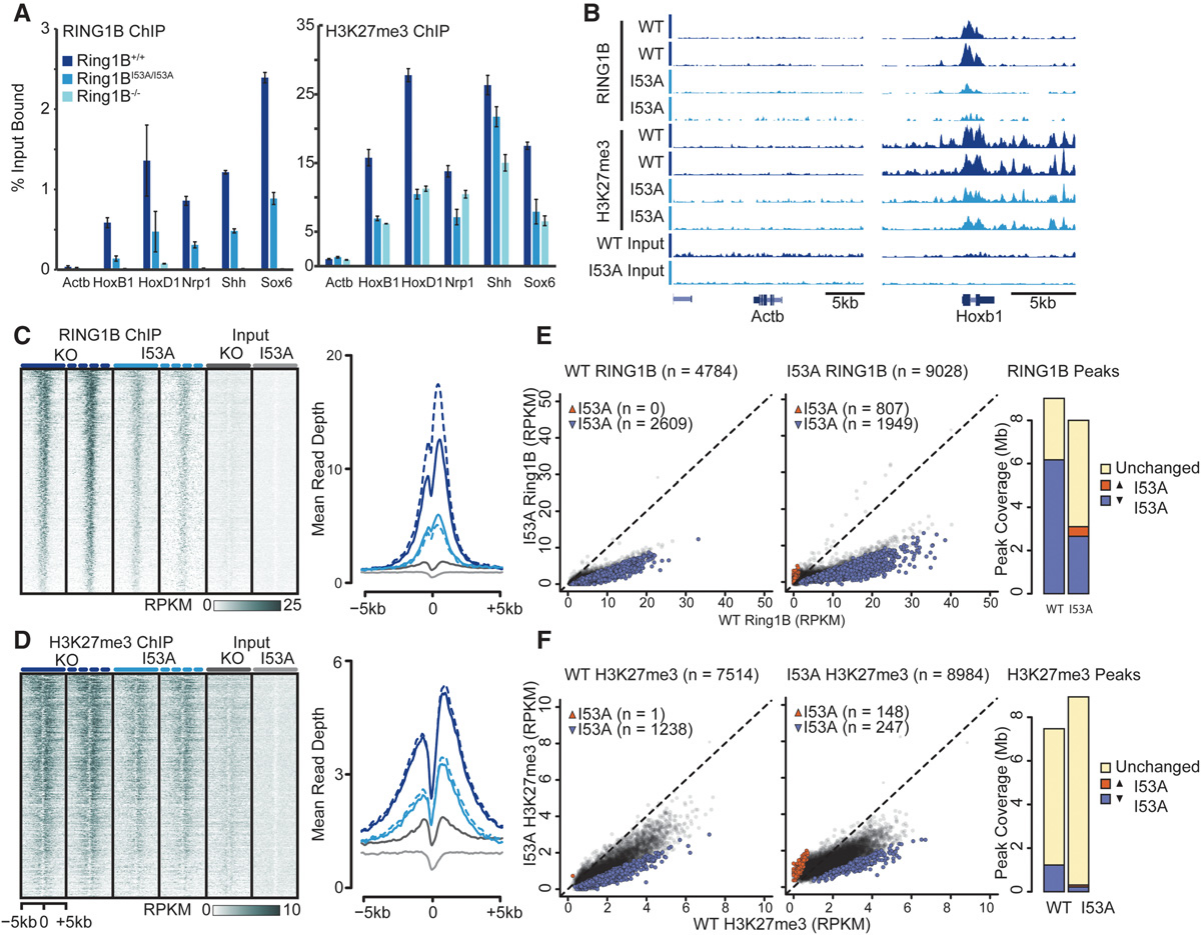
Mouse ESCs expressing catalytically inactive RING1B display impaired RING1B and H3K27me3 deposition. (*A*) RING1B and H3K27me3 levels (percentage input bound) for selected loci measured by ChIP-qPCR. (*B*) Genome browser screenshots showing normalized read depth for RING1B and H3K27me3 ChIP-seq in wild-type (WT) and *Ring1B*^*I53A/I53A*^ mESCs. (*C*,*D*) Heat maps depicting normalized ChIP-seq signal across RING1B-enriched TSSs (±5 kb) for RING1B (*C*) and H3K27me3 (*D*), ranked from highest to lowest ChIP-seq signal in wild-type mESCs. Average profiles are shown in the *right* panels. (*E*,*F*) Comparison of RING1B (*E*) and H3K27me3 (*F*) occupancy at RING1B-enriched regions in wild-type mESCs. Peaks were considered increased or decreased (red and blue spots, respectively; numbers given in parenthesis) if both replicates of *Ring1B*^*I53A/I53A*^ showed an at least twofold differential signal compared with that observed in both wild-type replicates. The *right* panels show the genomic size occupied by each category of enriched region.

In mammalian genomes, the placement of PRC2 has been suggested to occur primarily at CpG islands (Deaton and Bird 2011; Klose et al. 2013). The conventional model for PcG targeting to chromatin is then the hierarchical recruitment of PRC1 by the prior binding and activity of PRC2. However, it has been suggested that a reciprocal situation may occur, with PRC1-mediated H2AK119ub initiating PRC2 binding, providing a mechanism by which PRC1 and PRC2 may cooperatively reinforce their respective binding (Blackledge et al. 2014; Cooper et al. 2014; Kalb et al. 2014). To determine whether loss of RING1B catalytic activity has an impact on where H3K27me3 is deposited in the genome, we performed chromatin immunoprecipitation (ChIP) for RING1B and H3K27me3 in wild-type and *Ring1B*^*I53A/I53A*^ mESCs. Quantitative PCR (qPCR) revealed that RING1B is present at multiple canonical Polycomb target sites in *Ring1B*^*I53A/I53A*^ cells, albeit at reduced levels compared with wild type (Fig. 3A; Supplemental Fig. 3A). In *Ring1B*^*I53A/I53A*^, H3K27me3 levels at these loci were reduced to a level similar to that observed in *Ring1B*^−/−^ mESCs. To investigate whether this is true genome-wide, we performed high-throughput sequencing on the RING1B and H3K27me3 ChIP-enriched material (ChIP-seq). Manual inspection of normalized mapped ChIP-seq reads showed that the qPCR results were replicated in the sequencing data (Fig. 3B; Supplemental Fig. 3B). Analysis of all transcription start sites (TSSs) with RING1B enrichment in wild-type mESCs showed RING1B and H3K27me3 at the same sites in *Ring1B*^*I53A/I53A*^ but at reduced levels (Fig. 3C,D). These data, in combination with the maintenance of total RING1B protein levels in *Ring1B*^*I53A/I53A*^ mESCs (Fig. 1D), suggested that there might be a global redistribution of RING1B in these cells. Consistent with this, we found that RING1B ChIP-seq signal was lost from CGI TSSs and gained within gene bodies (Supplemental Fig. 4A). Although both RING1B- and H3K27me3-enriched regions in wild type typically had decreased signal in *Ring1B*^*I53A/I53A*^ cells (Fig. 3E,F), the absolute number of RING1B/H3K27me3-enriched regions identified in *Ring1B*^*I53A/I53A*^ was greater than that found in wild-type; we identified >5200 unique RING1B-occupied sites (Fig. 3E,F; Supplemental Fig. 4B). These ectopic “peaks” were at sites of low RING1B signal, showed a modest but significant (*P*-value of <1 × 10^−16^, Wilcoxon rank sum test) increase in ChIP-seq signal in the *Ring1B*^*I53A/I53A*^ mESCs (Supplemental Fig. 4B), and occurred preferentially within gene bodies (Supplemental Fig. 4C). To determine whether RING1B and H3K27me3 levels may be simply tracking transcription, we compared RING1B/H3K27me3 levels over rescued and nonrescued genes, and this revealed that the genes most up-regulated in *Ring1B*^*I53A/I53A*^ cells do indeed have a higher loss of H3K27me3 and RING1B ChIP signal (Supplemental Fig. 3C,D). Despite this, the abundance of sites with reduced H3K27me3/RING1B levels in *Ring1B*^*I53A/I53A*^ cells greatly exceeds the number of differentially expressed genes, suggesting that altered expression alone is not the principal driver of these chromatin changes.

While our data align with the model in which PRC1 enzymatic activity can direct PRC2 recruitment, we cannot easily discount the possibility that abrogated H3K27me3 deposition is due to an alternative deficit in the function of *Ring1B*^*I53A/I53A*^. Two lines of evidence suggest that this is not the case. First, there are no obvious problems with the composition of canonical PRC1 that contains RING1B^I53A^ (Fig. 1D; Illingworth et al. 2012). Moreover, despite a clear concordance between RING1B and H3K27me3 levels, interrogation of loci identified as invariant for RING1B occupancy between wild-type and *Ring1B*^*I53A/I53A*^ ESCs (Supplemental Fig 5) identified highly variable levels of H3K27me3 with no loss or gain of H3K27me3 as a group. Consequently, we believe that our data provide some support for the self-reinforcing recruitment model mediated in part by H2AK119ub.

### RING1B catalytic activity is dispensable for early mouse development

**Figure 4.**
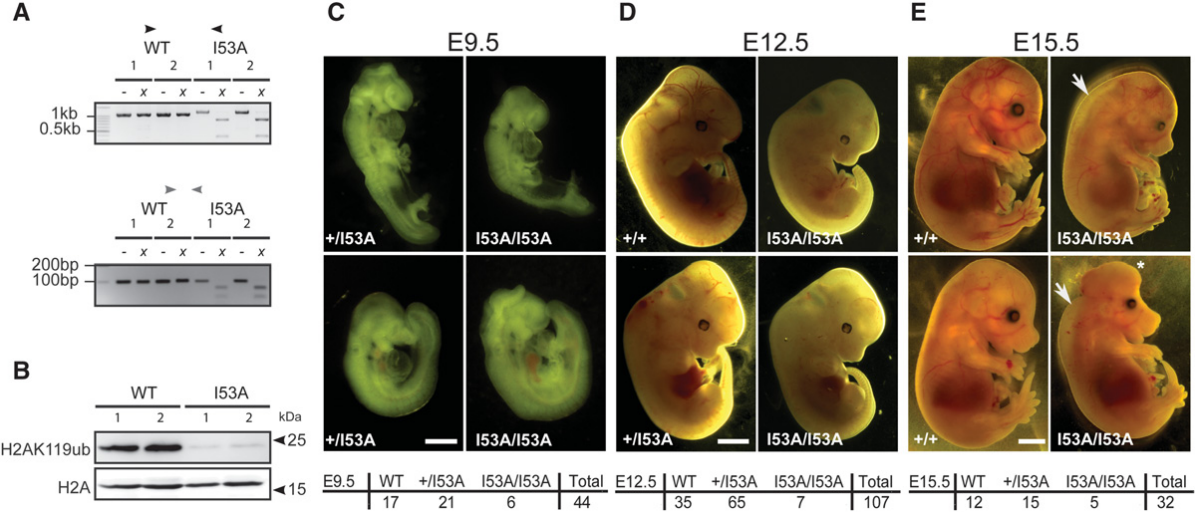
The E3 ubiquitin ligase activity of RING1B is not essential for early mouse embryo development. (*A*) Genotyping PCR performed on DNA prepared from E12.5 livers for exon 3-spanning (*top* panel) and exon 3 internal (*bottom* panel) primer sets (locations illustrated in Fig. 1A) show the expected undigested (−) and XbaI-digested (*x*) product sizes for both the wild-type (WT) and I53A alleles in E12.5 embryos. (*B*) Immunoblotting of acid-extracted histones from E12.5 wild type and I53A E12.5 placenta for H2A and H2AK119ub. (*C*–*E*) Photographs of embryos from a cross between *Ring1B*^+/*I53A*^ heterozygotes at E9.5 (*C*), E12.5 (*D*), and E15.5 (*E*). The *Ring1B* genotype is indicated *above* each panel, and the total number of embryos for all litters is tabulated *below*. Bars: E9.5 and E12.5, 0.5 mm; E15.5, 2 mm. (*C*) For E15.5 *Ring1B*^*I53A/I53A*^, swelling characteristic of edema and exencephaly are indicated (white arrows and asterisk, respectively).

Whereas RING1A is dispensable for embryonic development (del Mar Lorente et al. 2000), RING1B is essential for gastrulation (Voncken et al. 2003). To determine the in vivo role for the catalytic function of RING1B, we generated *Ring1B*^+/*I53A*^ mice from heterozygous knock-in mESCs. Correct targeting was validated in embryonic day 12.5 (E12.5) embryos using the PCR strategy illustrated in Figure 1A. Immunoblotting showed a major reduction in H2AK119ub levels in placentas of E12.5 *Ring1B*^*I53A/I53A*^ embryos when compared with wild type (Fig. 4A,B). Successive heterozygous matings did not yield live-born homozygous pups (98 *Ring1B*^+/+^, 147 *Ring1B*^+/*I53A*^, and 0 *Ring1B*^*I53A/I53A*^; χ^2^ = 88.2, *P* < 0.0001), suggesting that the E3 ligase activity of RING1B is required for full murine embryonic development. However, in contrast to the reported embryonic lethality of *Ring1B*^−/−^ by E10.5, we found that *Ring1B*^*I53A/I53A*^ embryos could complete gastrulation and develop to E15.5, albeit at sub-Mendelian frequencies (χ^2^ = 3.2, *P* = 0.20 at E15.5; χ^2^ = 19.6, *P* < 0.0001 at E12.5; χ^2^ = 5.6, *P* = 0.06 at E9.5) (Fig. 4C–E). No gross morphological abnormities or anterior–posterior patterning defects were seen at E9.5 or E12.5. At E15.5, two of the five *Ring1B*^*I53A/I53A*^ embryos that we recovered were developmentally retarded, but the morphology of the remaining three *Ring1B*^*I53A/I53A*^ embryos was largely normal (exemplified in Fig. 4E). All three of these E15.5 *Ring1B*^*I53A/I53A*^ embryos exhibited edema (Fig. 4E, arrows), which was never seen in any of the 27 control littermate embryos at this stage (Fisher's test, *P* < 0.01), suggesting some defects in development of the cardiovascular system. One of these three E15.5 *Ring1B*^*I53A/I53A*^ embryos exhibited exencephaly (Fig. 4E, asterisk). Interestingly, the co-occurrence of these two phenotypes is also seen in embryos deficient for the H3K27me3 demethylase KDM6A (Shpargel et al. 2012). It has been reported that the gastrulation stage lethality of *Ring1B*^−/−^ mice can be overcome by simultaneous loss of CDKN2A (Voncken et al. 2003). However, *Cdkn2a* expression remains up-regulated in *Ring1B*^*I53A/I53A*^ ESCs (Fig. 2D), and so the developmental rescue of gastrulation in *Ring1B*^*I53A/I53A*^ embryos may occur through a CDKN2A-independant mechanism.

We showed that catalytically inactive RING1B disrupts H3K27me3 deposition at target loci in ESCs, consistent with a model in which PRC1 and PRC2 cooperatively reinforce each other's binding, with the loss of PRC1 activity prompting some loss of H3K27me3, which in turn reduces PRC1 binding. We cannot exclude that reduced levels of H3K27me3 are not just a consequence of increased transcription in mutant cells (Riising et al. 2014). Despite this disruption of the epigenetic landscape, catalytically inactive RING1B is able to maintain near wild-type levels of gene expression compared with *Ring1B*-null ESCs and support embryonic development to an extent much greater than that reported for *Ring1B* knockout. Our findings support the notion that loss of RING1B E3 ligase activity and the consequent loss of most H2AK119ub only partially disrupt polycomb recruitment and function, consistent with the ability of ectopically expressed catalytically inactive RING1B to maintain chromatin compaction at polycomb target loci (Eskeland et al. 2010). Together with the importance of other PRC1 subunits in modulating higher-order chromatin structure (Grau et al. 2011; Isono et al. 2013), we suggest that the primary role for RING1B in gene repression and early embryonic development is structural rather than enzymatic.

## Materials and methods

### Generation of Ring1B^I53A/I53A^ and Ring1B^−/−^ mice

The targeting vector to knock in the I53A mutation into exon 3 of *Ring1B* (*Rnf2*) was generated by BAC recombineering (Liu et al. 2003). Briefly, a 129S7/AB2.2-derived BAC, bMQ291b2 (Adams et al. 2005), was modified using galK-positive/negative selection to introduce the I53A mutation and two silent restriction sites (SacI and XbaI) into exon 3 of *Ring1B* and a 10.1-kb region of the BAC (chromosome 1: 153,321,960–153,332,059; mm9) cloned into PL253 by gap repair. A floxed neomycin resistance cassette was then integrated into intron 3 (position chromsome 1: 153,323,749; mm9) in this gap-repaired plasmid using a mini targeting vector. The resulting plasmid was linearized and electroporated into E14TG2a ESCs (Joyner 2000), and G418-resistant clones were screened by PCR to identify correct targeting events. The neomycin resistance cassette was removed from correctly targeted clones by transient transfection of a Cre-expressing plasmid. *Ring1B*^+/*I53A*^ mESCs were injected into blastocysts to generate chimeric mice and backcrossed three times to C57BL/6 (Joyner 2000). A second round of targeting in *Ring1B*^+/*I53A*^ mESCs generated *Ring1B*^*I53A/I53A*^ mESCs.

*Ring1B*^−/−^ mESCs were generated using a similar strategy, except that a gap-repaired PL253 plasmid containing chromosome 1: 153,321,960–153,332,059 (mm9) and a wild-type version of *Ring1B* exon 3 was modified so that a lone LoxP site was introduced into intron 2 (chromosome 1: 153,324,264; mm9), and a floxed neomycin resistance cassette was introduced into intron 3 (chromosome 1: 153,323,749; mm9) using mini targeting vectors. Cre-mediated removal of the neomycin resistance cassette generated either a conditional knockout *Ring1B* allele with exon 3 flanked by LoxP sites or a *Ring1B*-null allele deleted for exon 3 that produces a transcript containing a premature STOP codon encoding a 42-amino-acid N-terminally truncated protein. Details of genotyping by PCR and cDNA sequencing are in Supplemental Table 2.

Generation and analysis of *Ring1B* mutant mice were performed under a UK Home Office project license (PPL 60/4424) with approval from an institutional ethics committee.

### Expression analysis

The Amino Allyl MessageAmp II with Cy3 kit (Ambion, AM1795) was used to produce cRNA using the manufacturer's protocol. Six-hundred nanograms of cRNA was fragmented and hybridized to a SurePrint G3 Mouse GE 8x60K microarray (Agilent, G4852A). After washing, the arrays were scanned using a NimbleGen scanner, and images were analyzed using Agilent Feature Extraction software. The resulting values were processed and analyzed using custom R scripts. Expression data were deposited in the Gene Expression Omnibus (GEO) repository (http://www.ncbi.nlm.nih.gov/geo) under accession number GSE69978. Details of qRT–PCR for verification of expression changes are in Supplemental Table 2. A full protocol is in the Supplemental Material.

### ChIP-seq

Libraries were prepared as previously described (Bowman et al. 2013) with modifications outlined in the Supplemental Material.

Sequence reads were trimmed (TrimGalore! version 0.2.7) to remove adapters (with the “-q 30” option used to remove low-quality bases with a PHRED score of <30 using Cutadapt version 1.2.1) and mapped to the mouse genome (mm10) using Bowtie 2.1.0 with the following arguments: “--local -D 20 -R 3 -N 1 -L 20 -i S,1,0.50.” SAM files were processed using HOMER version 4.3. HOMER tag directories were created using the makeTagDirectory tool with the options “-unique” and “-fragLength 150” and were used to create BedGraphs for visualization. All data output from HOMER analysis was normalized to 10 million mapped reads. BedGraphs were created using HOMER's makeUCSCfile tool with default options. Enriched regions were identified using the findPeaks tool from HOMER with the options “-style histone” and “-minDist 500” with the input sequences as controls. High-confidence enriched regions of at least 500 base pairs enriched in both replicates were identified. The HOMER analyzeRNA tool with the “-rpkm” option was used to quantify regions of interest. To generate heat maps, HOMER's annotatePeaks tool was used (options “-ghist” and “-hist 50”) to generate a matrix of RPKM (reads per kilobase per million mapped reads) values, which was processed using custom R scripts. Illumina sequencing data were deposited in the GEO repository (http://www.ncbi.nlm.nih.gov/geo) under accession number GSE69978.

Details of cell culture conditions, RNA extraction, cDNA synthesis, protein extractions, ChIP, and immunoblotting are provided in the Supplemental Material.

## Supplementary Material

Supplemental Material
