# Supplementary material for: The E3 ubiquitin ligase activity of RING1B is not essential for early mouse development
Source: Genes Dev. 2015 Sep 15;29(18):1897–902. doi: 10.1101/gad.268151.115 (PMC4579347; doi:10.1101/gad.268151.115)
Supplement: Supplemental Material [file supp_29_18_1897__index.html]

Supplemental Material 

# The E3 ubiquitin ligase activity of RING1B is not essential for early mouse development

## Supplemental Material

**Files in this Data Supplement:**

- Supp Material.docx
- Supp Table 1.xlsx
- Supp Table 2.docx
- Supp Table 3.docx
