# Supplementary material for: The E3 ubiquitin ligase activity of RING1B is not essential for early mouse development
Source: Genes Dev. 2015 Sep 15;29(18):1897–902. doi: 10.1101/gad.268151.115 (PMC4579347; doi:10.1101/gad.268151.115)
Supplement: Supplemental Material [file supp_29.18.1897_SuppMaterial.docx]

**Supplemental Figures**

**
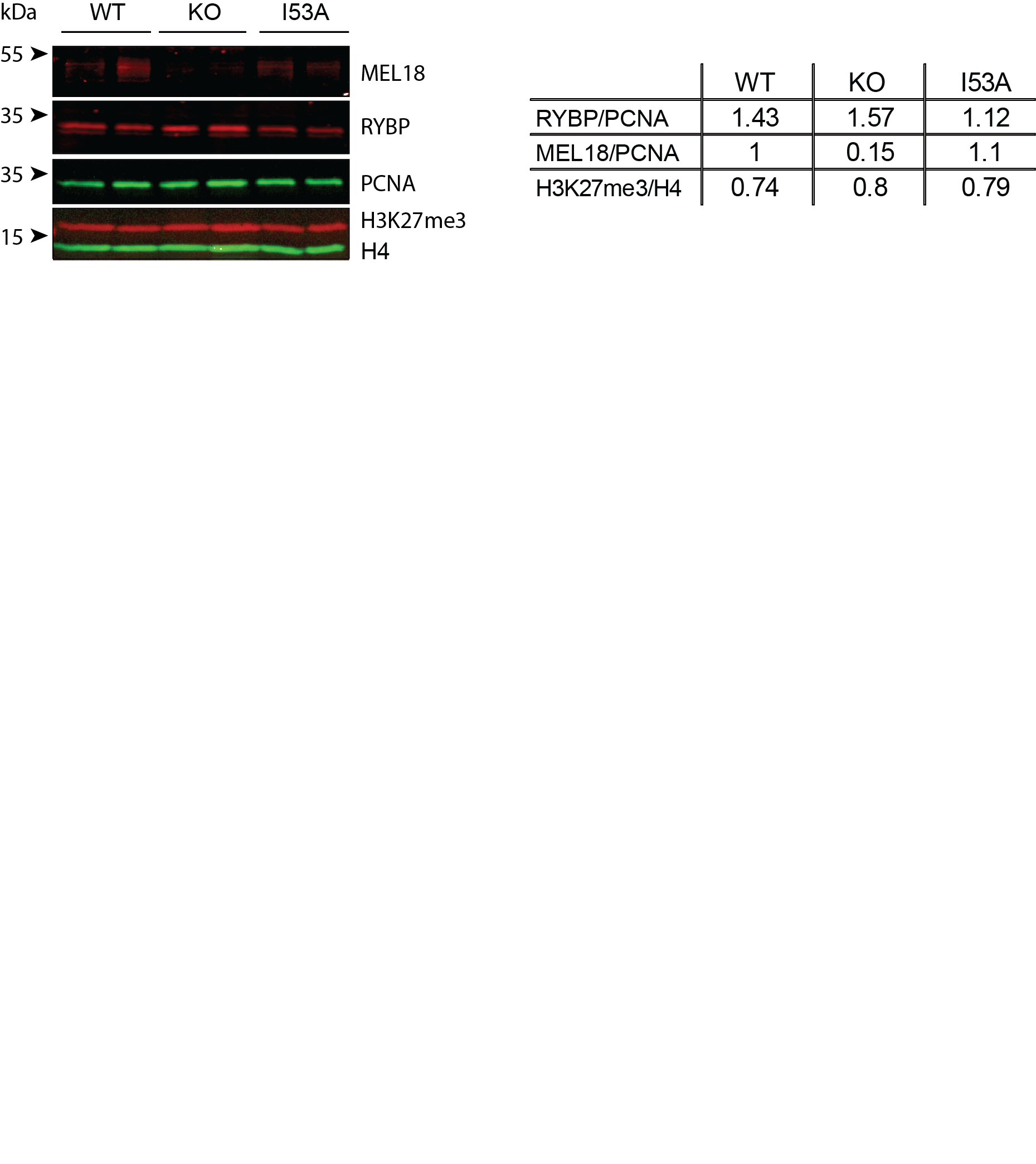
**

**Supplemental Figure 1. Quantitation of protein levels in WT, *Ring1B^I53A/I53A^* and *Ring1B^-/-^* mESC nuclear extracts**. Fluorescent immunoblots (left panel) and the mean signal quantification relative to the loading controls PCNA or H4 (right panel) from WT, *Ring1B^-/-^* (KO) and Ring1B^I53A/I53A^ (I53A) mESC nuclear extracts. Values represent the mean of 2 biological replicates.

**
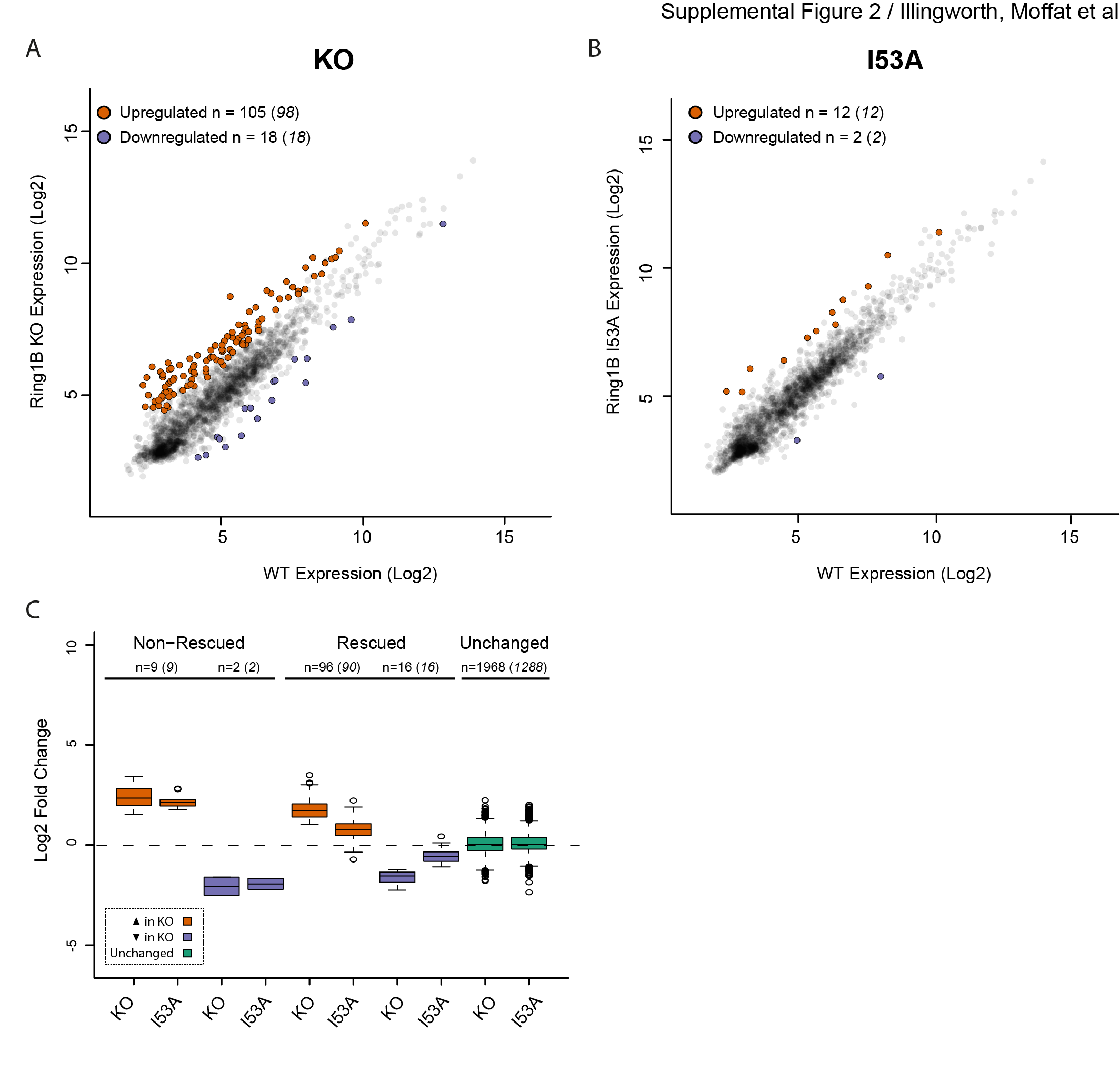
**

**Supplemental Figure 2. Expression analysis of RING1B target genes**. (A,B) Log2 expression values for genes enriched for RING1B at their TSS in WT vs. (A) *Ring1B^-/-^* mESCs and (B) *Ring1B^I53A/I53A^* mESCs (B) from expression microarrays. (A,B) Probes were considered to be upregulated (red) or downregulated (blue) if they had a Log2 fold change > 1 or < -1 respectively and a Benjamini-Hochberg corrected p-value < 0.05. The number of differentially expressed probes and the number of genes they represent (in parenthesis) are indicated. (C) Boxplots of Log2 fold expression changes for Ring1B^-/-^ vs. WT (KO) and Ring1B ^I53A/I53A^ vs. WT (I53A) for RING1B target genes with ‘Rescued’ and ‘Non-Rescued’ expression levels in Ring1B^I53A/I53A^ mESCs. The number of probes (genes in parentheses) is indicated for each subset.


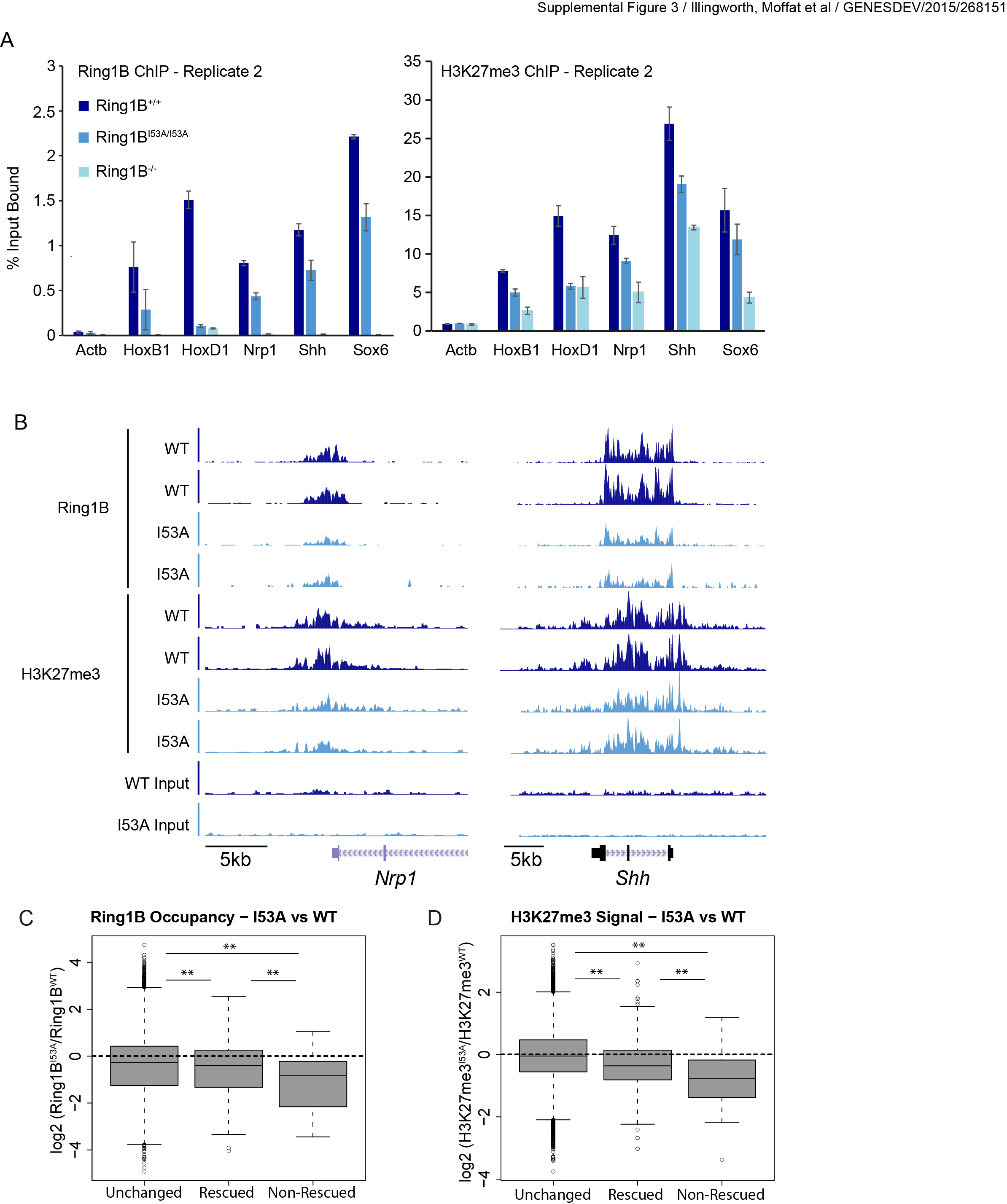
**Supplemental Figure 3.** Mouse ESCs expressing catalytically inactive RING1B display impaired RING1B and H3K27me3 deposition. (A) RING1B and H3K27me3 levels (relative % input bound) for selected loci measured by ChIP-qPCR. Data shown is a second biological replicate. (B) Genome browser screenshots showing normalised read depth for RING1B and H3K27me3 ChIP-seq in WT and Ring1B^I53A/I53A^ mESCs. (C,D) Boxplots depicting RING1B (C) and H3K27me3 (D) levels at TSSs, categorised based on their expression characteristics in mutant ESCs; upregulated in both Ring1B^-/-^ and Ring1B^I53A/I53A^ (Non-Rescued); upregulated in Ring1B^-/-^ but not Ring1B^I53A/I53A^ (Rescued); no change in expression (Unchanged).

**
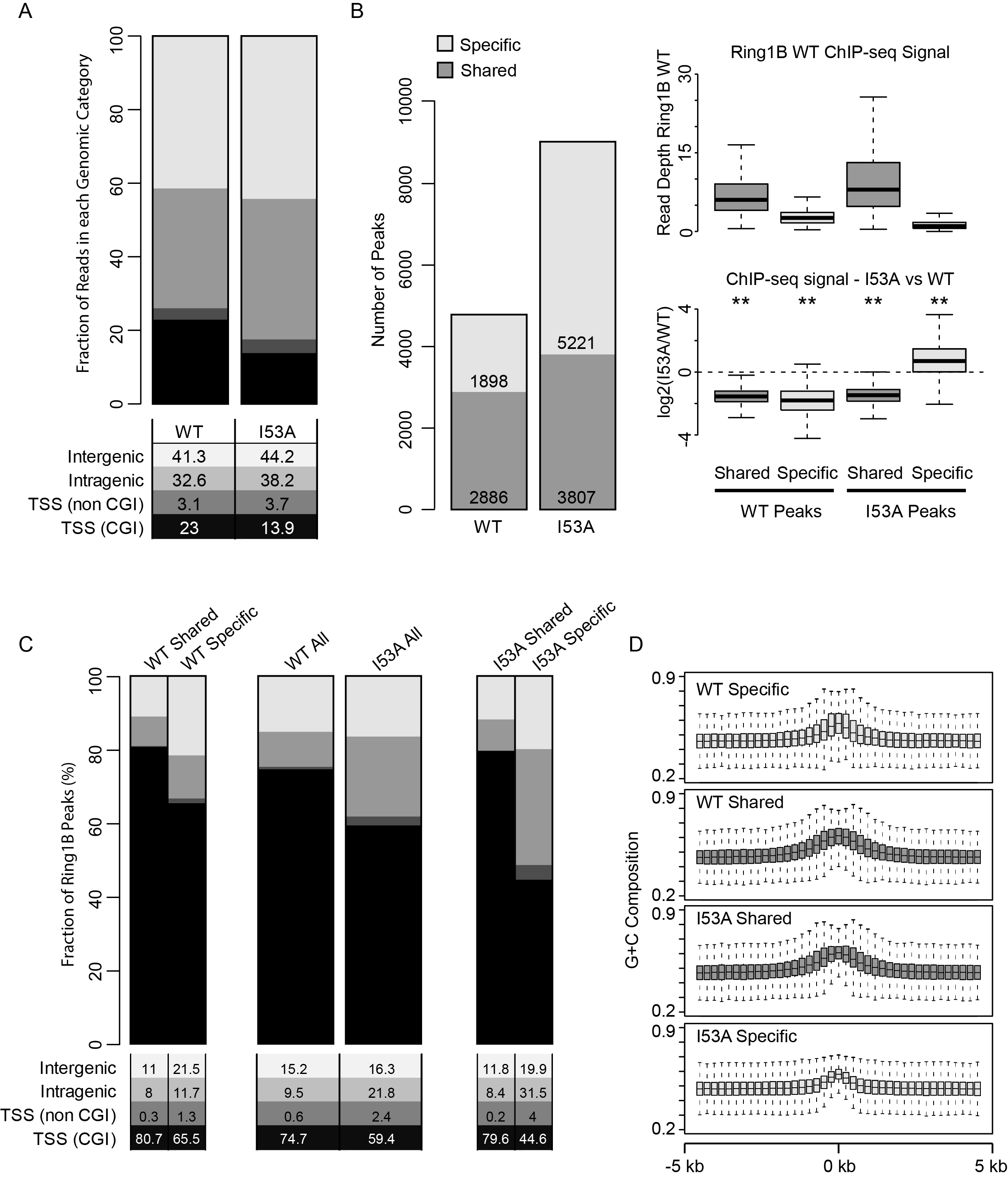
**

**Supplemental Figure 4. Characterisation of ectopic RING1B binding in Ring1B^I53A/I53A^ mESCs.** (A) Barplot showing the percentage of the total RING1B ChIP-seq signal in WT and Ring1B^I53A/I53A^ (I53A) mESCs which mapped to TSSs (CGI; +/- 5kb; black), TSSs (non-CGI; +/- 5kb; dark grey), gene bodies (Intragenic; grey bars) or non-coding regions (Intergenic; light grey). Percentages for each category as a fraction of total reads are tabulated below the barplot. (B) Barplot representing the number of RING1B ‘peaks’ identified in WT and Ring1B^I53A/I53A^ (I53A) mESCs separated into those which overlap (shared; grey) or are unique to (specific; light grey) each of the cell lines (left panel). The upper boxplot shows the RING1B ChIP-seq read depth in WT cells for each category and the lower boxplot shows the ratio of the read depth between the two cell lines (log2 (Ring1B^I53A/I53A^/WT)). Significance of differential RING1B occupancy was determined using a ‘Wilcoxon Rank Sum Test’ (**p value of <0.01). C) Barplot showing the genomic distribution of all RING1B peaks (central plot) or of these peaks further stratified into ‘shared’ and ‘specific’ for WT and Ring1B^I53A/I53A^ (right and left respectively). Figure composition as for panel (A). (D) GC base composition boxplots of 500 bp tiled windows (250 bp slide) across RING1B peaks for each of the 4 peak sets.

**
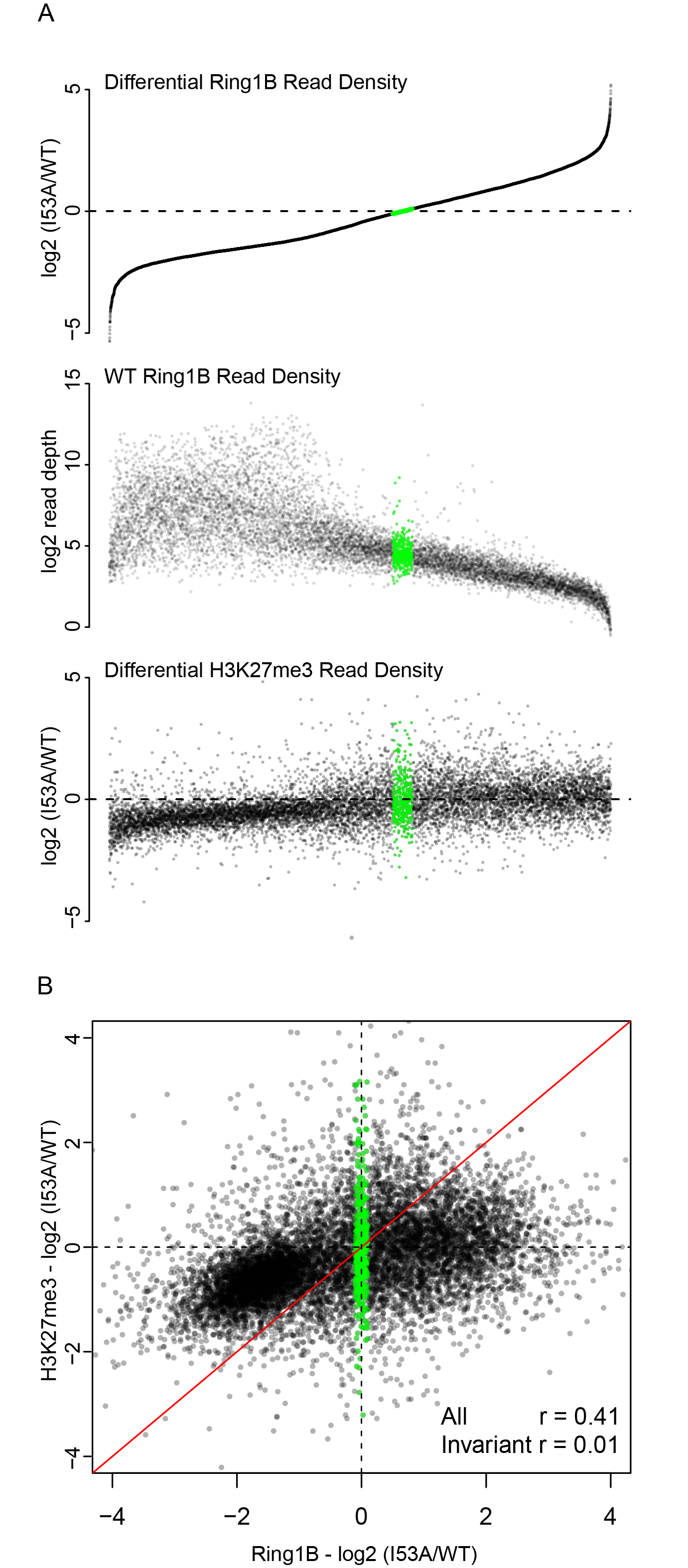
Supplemental Figure 5. Sites of invariant RING1B binding show variable levels of H3K27me3 deposition.** (A) Scatter plots showing the ratio of RING1B ChIP-seq signal (log2 (Ring1B^I53A/I53A^/WT); upper panel); the RING1B ChIP-seq signal in WT mESCs (middle panel); and the ratio of H3K27me3 ChIP-seq signal (log2 (Ring1B^I53A/I53A^/WT); lower panel). All plots are ranked by the level of differential RING1B occupancy from the largest loss to the largest gain in Ring1B^I53A/I53A^ cells relative to WT (left to right). Green spots represent genomic loci with invariant levels of RING1B occupancy between the two mESC lines (invariant refers to all values within the range of ≥ -0.1 and ≤ 0.1). (B) Scatter plot depicting differential RING1B occupancy (log2(I53A/WT)) vs differential H3K27me3 levels (log2(I53/WT)). Colouring as for panel (A). The legend provides the Pearson correlations scores (r) for all (All) and for RING1B invariant (Invariant) peaks as defined for (A).

**Supplemental Materials and Methods**

**Cell culture**

E14TG2a ESCs (129/Ola background) and the derivative *Ring1B^I53A/I53A^* and *Ring1B^-/-^* lines were cultured on 0.1% gelatin (Sigma G1890) coated Corning flasks in GMEM BHK-21 (Gibco 21710-025) supplemented with 10 % foetal calf serum (FCS; Sigma F-7524), 1,000 units/ml LIF, non-essential amino acids (Sigma M7145), sodium pyruvate (Sigma S8636), 2-β-mercaptoethanol (Gibco 31350-010), L-glutamine and penicillin/streptomycin.

**RNA extraction and cDNA synthesis**

Cells were washed in PBS and RNA extracted using the RNeasy Mini Kit (Qiagen 74104). 1 μg RNA was combined with 1 μl 10 mM dNTPs, 0.5 μl random 6mers (Promega C118A) in a 12 μl volume and incubated at 65 °C for 5 min, then snap chilled on ice. Reactions were combined with 4 μl 5X First Strand Buffer and 2 μl 0.1 M DTT (SuperScript II kit - Life Technologies 18064-014) and 1 μl RNasin (Promega N2515) and incubated at 25 °C for 2 min. 1 μl (200 units) SuperScript II enzyme were added and incubated at 25 °C for 10 min, 42 °C for 50 min and 72 °C for 15 min.

**Expression Analysis**

1 μg RNA, mixed with RNA standards (One Colour RNA Spike-In Kit; Agilent – 5188-5282) were labelled with cyanine 3 (Cy3) using the Amino Allyl MessageAmp™ II with Cy™3 kit (Ambion; AM1795). 600 ng of Cy3-labelled cRNA was fragmented at 60 °C for 30 min in 250 μl containing 1x Agilent fragmentation buffer and 2x Agilent blocking agent. 250 μl 2x Agilent hybridization buffer were added and hybridized to a SurePrint G3 Mouse GE 8x60K microarray (Agilent; G4852A) for 17 hours at 65 °C in a rotating hybridisation oven. After hybridisation, microarrays were washed 2x 1 min at room temperature with GE Wash Buffer 1, 2x 1 min with 37 °C GE Wash Buffer 2 (Agilent), then dried by brief centrifugation. Microarrays were scanned on a Nimblegen scanner at 2 μm resolution and 25 % gain to generate single channel TIFF images which were analysed with Feature Extraction Software (Agilent) using default parameters (protocol GE1_1105_Oct12 and Grid: 028005_D_F_20131202). The arithmetic mean was used for duplicate probes. The limma R/Bioconductor package was used for quantile normalisation across samples and to calculate fold-changes and p-values for differential expression using the empirical Bayes statistics for differential expression (eBayes) framework. Probes were assigned to genes by mapping to Ensembl transcripts, and identifying probes mapping with no more than one mismatch, deletion, or insertion. Probe sequences were input to RepeatMasker using the cross_match search engine (Speed/Sensitivity: slow; DNA source: mouse). Probes were only included subsequently if they mapped to transcripts from one gene and had < 50 % repetitive sequence. The Benjamini-Hochberg method was used to adjust p-values for multiple testing. Differential expression was defined as fold change > 2 or < 0.5 and an adjusted p-value of < 0.05. Three biological replicates were used per sample. Expression data were deposited in the GEO repository (http://www.ncbi.nlm.nih.gov/geo/) under the accession: GSE69978.

Details of quantitative RT-PCR for verification of expression changes are given in Supplemental Table 2.

**DNA Extraction for Genotyping**

Genomic DNA was extracted from mouse tails using "DNA releasy" as per the manufacturer’s instructions (Anachem) and from embryonic livers (E12.5) using Trizol reagent (Life Technologies, 15596-026). Briefly whole liver was dissolved in 500 μl of Trizol and 100 μl of chloroform was added. RNA was removed by discarding the aqueous phase obtained from centrifugation at 16,000 g for 10 min. 250 μl of Back Extraction Buffer (4 M guanidine thiocyanate; 50 mM sodium citrate; 1 M Tris pH 8.0) was added to the remaining organic phase which was then centrifuged at 16,000 g for 10 min. The DNA-containing aqueous phase was combined with the same volume of chloroform, centrifuged at 16,000 g, and the resulting aqueous phase treated with RNase cocktail (Life Technologies, AM2286) for 6 hours at 37 °C. Phenol:chloroform extraction and isopropanol precipitation was then used to obtain purified DNA.

**Nuclear and histone extracts**

*ESC Preparations*

Cell pellets were washed with PBS and resuspended in hypotonic buffer (10 mM Tris pH 8; 1.5 M MgCl_2_; 10 mM KCI; 0.5 mM DTT; 1X protease inhibitors) at 10^7^ cells/ml. Samples were kept on ice for 30 min with vortexing every 10 min, centrifuged at 4 °C at 3000 *g* for 15 minutes, and the pellets resuspended in RIPA buffer (50 mM Tris pH 7.5; 300 NaCl; 1 % NP40; 0.5 % sodium deoxycholate; 1X protease inhibitors).

For acid extraction of histones, nuclei were resuspended in 0.4 M HCl and placed on a rotating wheel at 4 °C overnight. After centrifugation at 13,000 rpm for 5 min at 4 °C, the supernatant was mixed with 0.5 volumes of TCA, then incubated on ice for 10 min. Precipitated histones were recovered by centrifugation, washed twice with ice-cold acetone and resuspended in RIPA buffer.

*Embryonic Preparations*

Embryonic placenta (E12.5) was dissolved in 500 μl of Trizol reagent (Life Technologies, 15596-026) and 100 μl of chloroform was added. RNA was removed by discarding the aqueous phase obtained from centrifugation at 16,000 g for 10 min. 250 μl of Back Extraction Buffer (4 M guanidine thiocyanate; 50 mM sodium citrate; 1 M Tris pH 8.0) was added to the remaining organic phase which was then centrifuged at 16,000 g for 10 min. The protein-containing organic phase was combined with 750 μl of isopropanol and centrifuged at 4 ºC for 30 min at 16000 g. The resulting pellet was washed twice by 20 min incubation in 0.3 M guanidine hydrochloride in 95 % ethanol on ice followed by centrifugation at 16,000 g. A final wash in ethanol was performed, and histones were isolated from the protein pellet by acid extraction by resuspending in HCl and processing as for HCl-resuspended ESC samples.

**Immunoblotting**

Nuclear extracts or histones were denatured at 95 °C for 5 min in NuPAGE LDS Sample Buffer (Life Technologies NP0008), separated by SDS-PAGE using a 4-12% NuPAGE Bis-Tris gel (NW04120BOX Life Technologies), then transferred to nitrocellulose membrane using an iBlot 2 at 20 V for 7 min (Life Technologies IB21001). Membranes were blocked with 5 % Bovine Serum Albumin (BSA) in TBST, then incubated with antibody in 1 % BSA/TBST overnight at 4 °C. Membranes were washed 3 x 10 min with TBST, then incubated overnight at 4 °C with an HRP-conjugated secondary antibody or LI-COR fluorescent secondary antibody. Membranes were washed with TBST 3 x 10 min, and detected as described previously for ECL ([Taylor et al. 2013](#_ENREF_2)) or imaged on The Odyssey® Sa Infrared Imaging System (LI-COR) and quantified using Image Studio Lite (v5.2; LI-COR). Antibodies are outlined in Supplemental Table 3.

**Cross-Linked Chromatin Immunoprecipitation**

2x10^7^ ESCs were washed twice in PBS, resuspended in 250 μl PBS and fixed by the addition of an equal volume 2 % methanol free formaldehyde/PBS (Thermo Scientific PN28906) at room temperature for 10 min. Fixation was stopped by the addition of glycine to 125 mM for 5 min. Cells were washed and pelleted in PBS prior to lysis. All subsequent buffers were supplemented with the following additives just prior to use: 0.2 mM PMSF, 1 mM DTT and 1x Protease inhibitors (Calbiochem, 539134-1SET). Cell pellets were resuspended in lysis buffer 1 (50 mM Tris-HCl pH 8.1, 10 mM EDTA and 20 % SDS) and incubated for 10 min at 4°C. Lysates were diluted 1:10 in ChIP dilution buffer (0.1 % Triton X-100, 2 mM EDTA, 150 mM NaCl, 20 mM and Tris-HCl pH 8.1) and sonicated using a chilled Bioruptor (Diagenode; 40x 1 min cycles of 30 sec on/ 30 sec off on ‘high’ setting at 4 °C). Sonicated extract was pre-cleared by centrifugation at 16000 *g* for 10 min at 4 °C and the supernatant transferred to a fresh tube and supplemented with BSA (25 μg/ml) and Triton X-100 (1 %). A sample was retained as input reference. Antibodies were pre-coupled to a 1:1 mix of protein A and G Dynabeads (Life Technologies; 10001D and 10004D respectively) at a ratio of 1 μg antibody: 30 μl dynabeads. 1.2x10^7^ and 6x10^6^ cell equivalents of lysate were added to 10 μg anti-Ring1B or 5 μg anti-H3K27me3 respectively and incubated for 10 h on a rotating wheel (antibody details in Supplemental Table 3). Bead-associated immune complexes were washed sequentially with wash buffers A, B and C for 10 min at 4 ºC on a rotating wheel followed by 2 washes in TE (wash buffer A – 1 % Triton X-100, 2 mM EDTA, 150 mM NaCl, 20 mM and Tris-HCl pH 8.1; wash buffer B – 1 % Triton X-100, 0.1 % Sodium-Deoxycolate, 0.1 % SDS, 1 mM EDTA, 500 mM NaCl, 20 mM and Tris-HCl pH 8.1; wash buffer C – 1 % NP40, 0.1 % Sodium-Deoxycolate, 1 mM EDTA, 250 mM LiCl, 20 mM and Tris-HCl pH 8.1). Chromatin was released by incubation with elution buffer (0.1 M NaHCO_3_ and 1 % SDS) for 15 min at 37 ºC followed by incubation at 65 ºC for 2 hours with RNaseA (20 mg/ml) and Tris pH 6.8 (100 mM). To degrade proteins and reverse cross-links, 50 μg proteinase K were added and incubated at 65 ºC overnight.

**Native Chromatin Immunoprecipitation**

3 x 10^6^ ESCs were centrifuged at 500 *g* for 3 min, washed twice in PBS and then resuspended in 200 μl of NBA buffer (85 mM NaCl, 5.5 % Sucrose, 10 mM TrisHCl pH 7.5, 0.2 mM EDTA, 0.2 mM PMSF, 1 mM DTT, 1x Protease inhibitors (Calbiochem, 539134-1SET)). Cells were lysed by the addition of an equal volume of NBA + 0.1 % NP40 and incubated on ice for 3 min. Nuclei were pelleted at 2000 *g* for 3 min at 4 ºC, then washed with NBR buffer (85 mM NaCl, 5.5 % Sucrose, 10 mM TrisHCl pH 7.5, 3 mM MgCl_2_, 1.5 mM CaCl_2_, 0.2 mM PMSF and 1 mM DTT) and pelleted at 2000 *g* for 3 min at 4 ºC. Nuclei were resuspended (1.2x10^7^ nuclei/ml) in NBR supplemented with RNaseA (20 μg/ml) and incubated at 20 ºC for 5 min. Chromatin was fragmented for 10 min at 20 ºC using 0.133 U/μl microccocal nuclease (Boehringer units; SigmaAldrich - N3755-500UN; titrated to give predominantly mono-nucleosomes). Digestion was stopped with the addition of an equal volume of STOP bufffer (215 mM NaCl, 10 mM TrisHCl pH 8, 20 mM EDTA, 5.5 %, Sucrose, 2 % TritonX 100, 0.2 mM PMSF, 1 mM DTT, 2X Protease Inhibitors) and digested nuclei left on ice overnight to release soluble, fragmented chromatin. Chromatin was pre-cleared by centrifugation at 12,000 *g* for 10 min at 4 ºC and the soluble chromatin (supernatant) transferred to a fresh tube. 10 % of the released chromatin was retained as input and the remainder incubated for 3 h at 4 ºC on a rotating wheel with 3 μg of anti-H3K27me3 antibody pre-coupled to protein A dynabeads as described above (Life Technologies; 10002D). Immune complexes bound to beads were washed 5x with wash buffer 1 (150 mM NaCl, 10 mM TrisHCl pH 8, 2 mM EDTA, 1 % NP40 and 1 % sodium deoxycholate) and twice in 1x TE at 4 ºC on a rotating wheel for 10 min. Chromatin was released from the beads by incubation with 0.1 M NaHCO_3_/ 1 % SDS for 15 min at 37 ºC followed by the addition of proteinase K (100 ug/ml) and Tris pH 6.8 (100 mM) and incubation at 55 ºC overnight. For both native and cross-linked ChIP, Dynabeads were removed using a magnetic rack and the chromatin purified using MinElute PCR Purification columns (Qiagen) according to manufacturer’s instructions.

Details of quantitative PCR for verification of ChIP data are given in Supplemental Table 2.

**ChIP-seq Library Preparation**

Libraries were prepared as previously described ([Bowman et al. 2013](#_ENREF_1)) with the following modifications: No purification was performed between the A-tailing and ligation reactions; instead the ligation was supplemented with ligation reagents (400 U of T4 DNA ligase (NEB), 1x buffer 2 (NEB), 7.5 % PEG-6000, 1 mM ATP and 13.3 nM of annealed Illumina adaptors (AU) and incubated at 25 °C for 90 min. Size selection following the ligation and PCR steps were performed with 1x and 0.8x reaction volumes of Agencourt AMPure XP beads respectively (Beckman Coulter - A63880). Six samples were multiplexed per Hi-seq lane and sequenced by the National High-throughput DNA Sequencing Centre in Denmark.

**References**

Bowman SK, Simon MD, Deaton AM, Tolstorukov M, Borowsky ML, Kingston RE. 2013. Multiplexed Illumina sequencing libraries from picogram quantities of DNA. *BMC Genomics* **14**: 466.

Taylor GC, Eskeland R, Hekimoglu-Balkan B, Pradeepa MM, Bickmore WA. 2013. H4K16 acetylation marks active genes and enhancers of embryonic stem cells, but does not alter chromatin compaction. *Genome Res* **23**: 2053-2065.
