# Supplementary material for: The E3 ubiquitin ligase activity of RING1B is not essential for early mouse development
Source: Genes Dev. 2015 Sep 15;29(18):1897–902. doi: 10.1101/gad.268151.115 (PMC4579347; doi:10.1101/gad.268151.115)
Supplement: Supplemental Material [file supp_29.18.1897_SuppTable_2.docx]

**Supplemental Table 2.**

For genotyping, PCR reactions comprising 20 ng genomic DNA, 10 μl 1 μM forward and reverse primer mix, 25 μl 2x DreamTaq Green PCR Master Mix (Life Technologies K1081) in 50 μl were amplified at 95°C for 30sec, followed by 35 cycles of 95°C 30 sec, 60°C 30 sec and 72°C 90 sec and a final extension of 72 °C for 5 min. For cDNA sequencing, 2 μl cDNA, 2.5 μl 10 μM forward and reverse primer mix (Ring1B_F1R5), 10 μl 5X Phusion HF buffer (NEB M0530S), 1 μl 10mM dNTPs, 0.5 μl Phusion High Fidelity Polymerase (1U) in 50 μl were amplified as above. PCR products were purified using QIAquick PCR Purification columns (Qiagen) and sequenced on a 3730 genetic analyser (Life Technologies).

Amplicon Sizes (primer sequences below):

Exon 3 internal primers: WT – 100bp; I53A – 100bp and KO – No product. *XbaI* digests I53A product only.

Exon 3 spanning primers: WT – 1072bp; I53A – 1173bp and KO – 489bp. *XbaI* digests I53A product only.

Quantitative PCR was performed on a Roche LightCycler 480 using the SYBR Select Master Mix (Life Technologies 4472908). PCRs were cycled as follows: 50 °C for 2 min, 95 °C for 2 min followed by 50 cycles of 95 °C/15 sec then 60 °C/ 1 min.

For transcript analysis, a standard curve for each primer set was obtained using a mix of each of the cDNAs. The relative expression of each sample was measured by the Lightcycler software and normalized to the mean for *Gapdh* from each of the 3 biological replicates. The arithmetic mean values and standard deviations for each primer were normalised to the WT mESC values.

To quantitate ChIP enrichment, a standard curve for each primer set was obtained using a 4 step 5 fold serial dilution of sonicated WT genomic DNA. Standard curves were used to calculate the IP/Input ratio using triplicated PCR reactions for each of the IP and input samples (inputs were set as a defined proportion of the starting material). Two biological replicates were performed for each ChIP-qPCR.

|  | **Primer Name** | **Forward Primer** | **Reverse Primer** |
| --- | --- | --- | --- |
| **Ring1B Genotyping** | Ring1B Exon3 Spanning | GTCCCAGCACCACATAAACC | CCTGATGCGCTTCATACTCA |
|  | Ring1B Exon3 Internal | AGATGGCTTGGAAATTGTGGT | CTCCTTTGTAGTCATGGTGTTCT |
|  |  |  |  |
| **Ring1B I53A Genotyping (Mice)** | I53A_Mouse_Geno | CTTGCCTTGGGTTTCTATGC | CCTGATGCGCTTCATACTCA |
|  |  |  |  |
| **Ring1B cDNA Sequencing** | Ring1B_F1R5 | TGATTCTCGAGTCTCGCTCC | ACAGTGGCATTGCCTGAAGT |
|  |  |  |  |
| **qRT PCR** | Gapdh_2 | TGCGACTTCAACAGCAACTC | CTTGCTCAGTGTCCTTGCTG |
|  | Ring1B_F4R5 | ACGGTGCCAGTGAGATTGAG | ACAGTGGCATTGCCTGAAGT |
|  | Oct4_2 | TCTGTTCCCGTCACTGCTC | TGTCTACCTCCCTTGCCTTG |
|  | Pcgf1_1 | CCGACTAATGCTAAATCCAC | GGGACAGCCATAGCTGTTTC |
|  | Pcgf2_1 | ATTTGGAGTCTCCCTCGACC | AACTTGAACAGGATCAGGCG |
|  | Rybp_1 | ACGAAGGCTTTTGGGATTGT | AGCTGAGAATTGATGCGAGG |
|  | Ezh2_1 | GAACTGTAGCATTCAGCGGG | TTTGATAAAGATGCCCCAGC |
|  | Ring1A | AGAATGCCAGCAAAACGTG | CGCAATCTCTGTACCATCCA |
|  | Tex19.1 | AAAATGGGCCACCCACATCTC | CCACTGGCCCTTGGACCAGAC |
|  | Cdkn2a_1 | AGAGCGGGGACATCAAGAC | GTTCCCAGCGGTACACAAAG |
|  | Hoxb13 | CATTCTGGAAAGCAGCGTTTGCAG | GATAACTTGTTGGCTGCATACTCC |
|  | Hoxd10 | GTGCAGGAGAAGGAAAGCAAAG | TAACGCTCTTACTGATCTCTAGGC |
|  |  |  |  |
| **ChIP qPCR** | Actb_pro_2 | GGCTGCAAAGAGTCTACACG | TCACTCAGAACGGACACCAT |
|  | Hoxb1_pro | TTAGCCCATTGGCCTGGGAGAGAT | TGAAGCTTGAGCTTGAGCCCATGGCCCG |
|  | Hoxd1_pro | GAGTAACTTGACCTTCTCAGAG | ATTGCGGGAGAAAGGCAGGGAAG |
|  | Nrp1_3 | CGGAGAGAAACTTGTGTCTC | GCTCTGCCAGTGATTGCCTG |
|  | Shh_3 | GGAGAGCTTGTGAGACAGGC | CGGCTTGGCCAATCAGATGC |
|  | Sox6_3 | GCATTTCCCGTCGTGCAACA | CCTTCGCCTCTTGTCTCATC |
