# Supplementary material for: The E3 ubiquitin ligase activity of RING1B is not essential for early mouse development
Source: Genes Dev. 2015 Sep 15;29(18):1897–902. doi: 10.1101/gad.268151.115 (PMC4579347; doi:10.1101/gad.268151.115)
Supplement: Supplemental Material [file supp_29.18.1897_SuppTable_3.docx]

Supplemental Table 3.

Details of antibodies used for immunobolotting and ChIP

| **Antibody** | **Usage** | **Dilution for Western Blot** | **Company** | **Catalogue Number** |
| --- | --- | --- | --- | --- |
| RING1B (Rnf2) | Western/ChIP-PCR | 1:500 | LSBio | C179401 |
| RING1B (Rnf2) | ChIP-seq | NA | MBL | D139-3 |
| H3K27me3 | Western (LI-COR)/ChIP | 1:2000 | Millipore | 07-449 |
| MEL18 (PCGF2) | Western | 1:500 | Santa Cruz | sc10744 |
| H3K36me3 | Western | 1:2000 | Abcam | AB9050 |
| H2A | Western | 1:500 | Millipore | 07-146 |
| H2AK119ub | Western | 1:500 | Millipore | 05-678 |
| RYBP (DEDAF) | Western (LI-COR) | 1:1000 | Millipore | AB3637 |
| PCNA | Western (LI-COR) | 1:2000 | SCBT | SC56 |
| IRdye 680 LT | Western (LI-COR) | 1:15000 | LI-COR | 925-68021 |
| IRdye 800 CW | Western (LI-COR | 1:15000 | LI-COR | 926-32210 |
